# Chromatin and Nuclear Dynamics in the Maintenance of Replication Fork Integrity

**DOI:** 10.3389/fgene.2021.773426

**Published:** 2021-12-14

**Authors:** Jack Wootton, Evi Soutoglou

**Affiliations:** Genome Damage and Stability Centre, School of Life Sciences, University of Sussex, Brighton, United Kingdom

**Keywords:** DNA replication, chromatin, DNA repair, nucleus, DNA damage

## Abstract

Replication of the eukaryotic genome is a highly regulated process and stringent control is required to maintain genome integrity. In this review, we will discuss the many aspects of the chromatin and nuclear environment that play key roles in the regulation of both unperturbed and stressed replication. Firstly, the higher order organisation of the genome into A and B compartments, topologically associated domains (TADs) and sub-nuclear compartments has major implications in the control of replication timing. In addition, the local chromatin environment defined by non-canonical histone variants, histone post-translational modifications (PTMs) and enrichment of factors such as heterochromatin protein 1 (HP1) plays multiple roles in normal S phase progression and during the repair of replicative damage. Lastly, we will cover how the spatial organisation of stalled replication forks facilitates the resolution of replication stress.

## 1 Introduction

Faithful replication of the genome is important to allow successful inheritance of genetic material from parental to daughter cells in all organisms. Eukaryotic DNA replication is preceded in G1 phase with the licencing of pre-replicative complexes (pre-RC), containing head-to-head dimers of minichromosome maintenance (MCM) helicase, to replicative origins throughout the genome in an origin recognition complex (ORC)-dependent manner (Parker et al., 2017). After the G1 to S phase transition, these pre-RCs are activated in a process called origin firing, which is highly dependent on Dbf4-dependent kinase (DDK) cyclin-dependent kinase (CDK) activity (Boos and Ferreira, 2019). Origin firing does not happen simultaneously across the genome; instead, different regions are replicated at different stages throughout S phase and this process is coordinated by various factors such as the local chromatin environment and sub-nuclear localisation. Control of origin firing is vital to ensure coordinated replication of the entire genome (Boos and Ferreira, 2019). Upon origin firing, Cdc45, and GINS are recruited to MCM to form the active CMG helicase which unwinds DNA into single stranded DNA (ssDNA) in a bidirectional manner, forming two replication forks (Burgers and Kunkel, 2017). Replication then occurs from these forks and DNA in synthesised in a semi-conservative manner. Due to the unidirectionality of the CMG helicase and replicative polymerases, the leading strand is synthesised continuously by polymerase epsilon (Polε) and the lagging strand is synthesised in discontinuous Okazaki fragments by polymerases alpha (Polα) and delta (Polδ) (Burgers and Kunkel, 2017).

Throughout S phase, replication forks may be challenged by various sources of replication stress that stall DNA synthesis through a variety of mechanisms. Some endogenous sources include challenging secondary DNA structures within repetitive sequences, barriers to replisome movement such as torsional stress or DNA-protein crosslinks (DPCs) and collisions with the transcription machinery (Zeman and Cimprich, 2014). Importantly, the high levels of endogenous replication stress observed in cancer cells are exploited to develop novel anti-tumour drugs (Ubhi and Brown, 2019). In addition, several exogenous agents can be used experimentally to induce replication stress; for example, hydroxyurea (HU) stalls replication by depleting the cellular supply of deoxyribonucleotides (Bianchi et al., 1986). In the event of replication stress, the cell coordinates a variety of pathways to maintain genome stability and ensure the completion of replication.

These processes occur in a highly organised genome. The genome is spatially arranged in a hierarchical manner from nuclear compartments and chromosome territories (CTs) to topologically associated domains (TADs) and nucleosomes with a range of chromatin states (Gibcus and Dekker, 2013). Genome-wide chromosome conformation capture methods (such as Hi-C) are important tools to delineate how different regions of chromatin interact with each other, allowing the identification of TADs: loops of chromatin with borders that restrict the activity of regulatory elements between different TADs (Dixon et al., 2012; Li et al., 2018). In addition, genomic regions are identified according to their transcriptional status as either A or B compartments. A compartments are transcriptionally active, open and gene-rich whereas B compartments are transcriptionally silent, compact and gene-poor (Gibcus and Dekker, 2013). At the smaller scale, genetic material is packaged into units called nucleosomes comprised of DNA wrapped around a histone octamer of two H2A-H2B dimers and two histone H3-H4 dimers which are connected by H1-bound linker DNA (Luger et al., 2012). The local chromatin environment defined by composition of these nucleosomes, histone post-translational modifications (PTMs) and enrichment of non-histone proteins can affect the levels of transcription and chromatin compaction. In this sense, chromatin is categorised into relaxed, transcriptionally active euchromatin and compact, transcriptionally inactive heterochromatin. For example, histone marks enriched within active chromatin include methylated H3K4 and acetylated H3K9 and inactive marks include trimethylated H3K9 and H3K27 (Kouzarides, 2007). Regions of chromatin can also be localised within specific sub-nuclear compartments; for example, the nuclear/nucleolar periphery, nuclear pores, within nucleoli and in the nuclear interior.

Other than transcriptional regulation, genome organisation has major regulatory roles in the repair of DNA damage and in faithful DNA replication. In this Review, we will outline how the organisation of the genome, ranging from higher order spatial arrangement to the local chromatin environment, impacts the complex process of replication and the response to replication stress.

## 2 How Chromatin Affects Unperturbed Replication

### 2.1 Replication Timing

#### 2.1.1 Higher Order Compartments and Nuclear Location

The spatial positioning of specific genomic regions greatly influences their replication timing (RT). This region-specific replication timing program is established during early G1 phase during a period called the timing decision point (TDP), coinciding with the restoration of genome organisation following cell division (Dimitrova and Gilbert, 1999; Dileep et al., 2015). Furthermore, TADs identified through mapping of the entire genome in several cell types correlate with the basic units of replication timing known as replication domains (RD); therefore, the global organisation of the genome contributes to RT at a greater level than the activation of individual replication origins (Pope et al., 2014). Notably, early and late replicating regions correlate with transcriptionally active A and silent B compartments, respectively, and this correlation can be followed even with resolution of nuclear sub-compartments (Ryba et al., 2010; Yaffe et al., 2010; Rao et al., 2014). Histone modifications enriched at specific nuclear compartments involved in transcriptional activation/repression also contribute to this control of replication timing (see below). Higher order reorganisation of the genome during mouse stem cell differentiation, which is coordinated with changes in transcription and cell fate determination, is also associated with changes in the RT program (Hiratani et al., 2008, 2010). Recently it was shown that reorganisation of the TADs is important in modulating origin firing efficiency (Li et al., 2018). During G1 phase, TADs can be rearranged by displacement of CTCF and chromatin decompaction mediated by transcription, which results in the relocation of replication origins to the TAD boundaries. At the spatial boundary of TADs, origin firing is more efficient due to the presence of proliferating cell nuclear antigen (PCNA) clusters that facilitate replication (Li et al., 2021). This describes a mechanism by which transcription plays a role in the regulation of replication initiation, a phenomenon described in multiple studies (Blin et al., 2019; Chen et al., 2019; Li et al., 2021).

**FIGURE 1:**
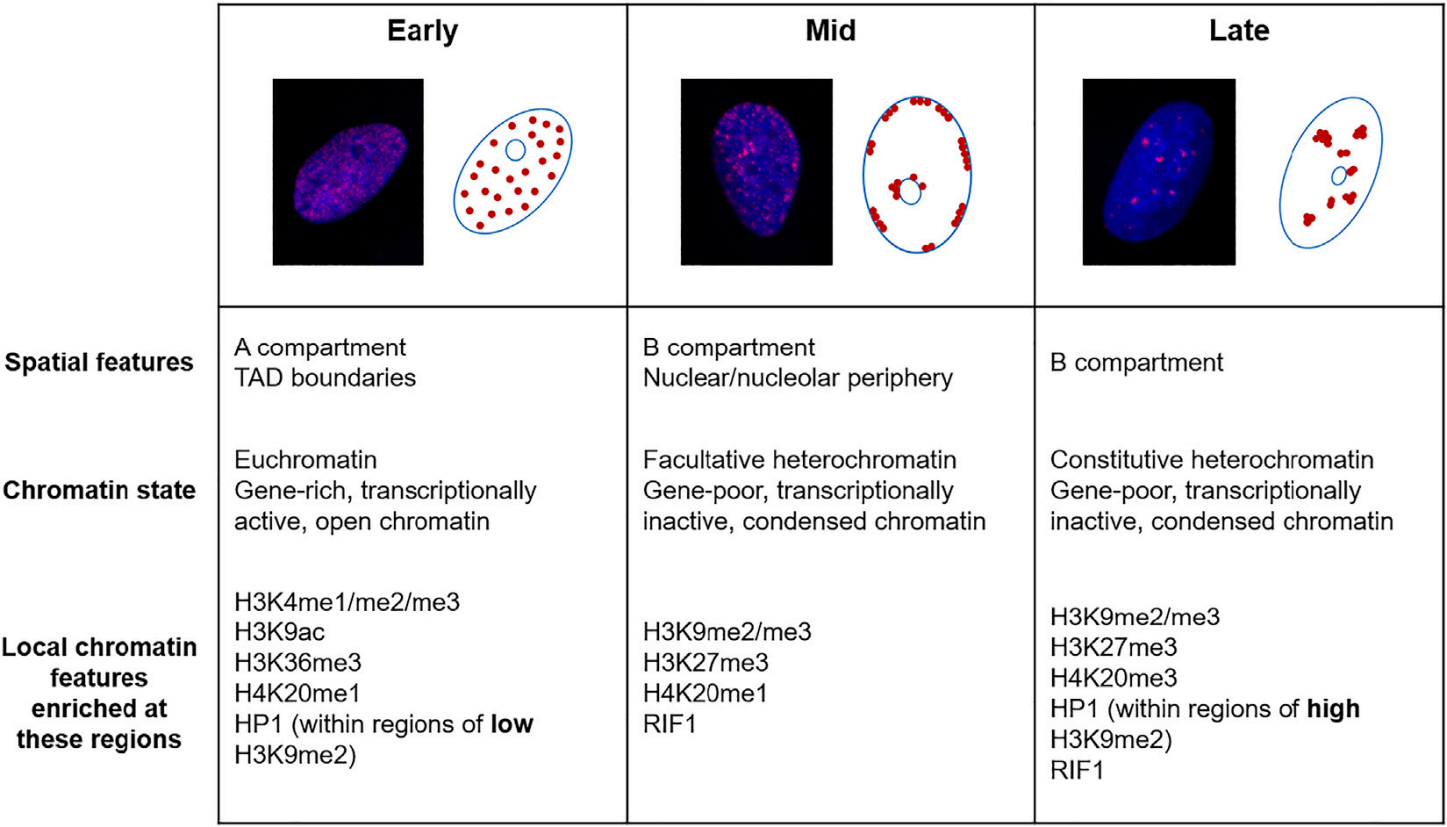
The replication timing of the eukaryotic genome. Fluorescence microscopy images of U2OS cells labelled with 5-ethynyl-2′-deoxyuridine (EdU, red) and DAPI (blue) and schematics showing the three distinguishable sub-phases of replication; early, mid and late. Below are descriptions of the chromatin replicated in each of these sub-phases.

In mammalian cells, the progression of S phase follows a distinct pattern, where euchromatin is replicated in early S phase, facultative heterochromatin at the nuclear/nucleolar periphery in mid S phase and constitutive heterochromatin in late S phase (Heinz et al., 2018) (Figure 1). Some heterochromatic regions of the genome, known as lamina-associated domains (LADs) are associated with the nuclear periphery and bind to the nuclear envelope via lamin proteins. These regions are generally replicated during mid-S phase, gene-poor and categorised under the transcriptionally repressed B compartment. In support of the nuclear periphery environment playing roles in replication timing, artificial targeting of mouse chromocentres containing constitutive heterochromatin to the nuclear periphery advances their replication timing from late to mid-S phase (Heinz et al., 2018). It has been hypothesised that the nuclear periphery contains the replication factors required for origin firing during mid S phase and therefore provides an environment where artificially tethered chromatin is replicated during this time. This advance in replication timing is not due to *de novo* deposition of facultative heterochromatin marks following artificial tethering, although constitutive histone marks are progressively lost at these regions following subsequent divisions (Heinz et al., 2018).

An important factor that connects genome organisation with the replication timing program is RIF1, a protein mainly involved in maintaining the late replication status of specific genomic regions (Hayano et al., 2012; Yamazaki et al., 2012; Peace et al., 2014; Foti et al., 2016; Hafner et al., 2018; Gnan et al., 2021). Specifically, RIF1 interacts with the nuclear lamina and plays a key role in regulating the replication timing of the LADs (Roux et al., 2012; Foti et al., 2016). Recruitment of RIF1 to RIF1 associated domains (RADs) leads to chromatin reorganisation of these regions and delays the replication timing through regulating origin firing (Yamazaki et al., 2012; Davé et al., 2014; Hiraga et al., 2014; Hiraga et al., 2017; Foti et al., 2016; Alver et al., 2017). This control of replication origin firing by RIF1 is highly dependent on its conserved interaction with protein phosphatase 1 (PP1) (Davé et al., 2014; Hiraga et al., 2014; Hiraga et al., 2017; Mattarocci et al., 2014; Alver et al., 2017; Gnan et al., 2021). Mechanistically, recruitment of PP1 to replication origins by RIF1 reverses the phosphorylation of the MCM complex by DDK, a modification that is required for initiation of replication, and therefore prevents premature replication at specific genomic loci (Davé et al., 2014; Hiraga et al., 2014).

#### 2.1.2 Link Between Histone Modifications and Replication Timing

In both mouse and human cell types, there is a broad correlation between early replicating regions with “active” chromatin marks such as methylated H3K4, H3K36me3, H4K20me1 and acetylated H3K9 but not “repressive” marks including di- and trimethylated H3K9 and H3K27me3 (Hiratani et al., 2008; Yokochi et al., 2009; Ryba et al., 2010; Picard et al., 2014) (Figure 1). In one study in human cells, the strongest correlation between later replication and repressive histone marks was detected with H3K9me2, which is commonly enriched at the nuclear periphery (Ryba et al., 2010).

In mouse embryonic stem cells (mESCs), the degree of DNA methylation affects replication timing through affecting histone modifications particularly at pericentric heterochromatin (Jørgensen et al., 2007a; Takebayashi et al., 2021). Partial loss of DNA methylation (Dnmt1 single knockout), as well as loss of other repressive chromatin modifiers, results in earlier replication of pericentric major satellite repeats (Jørgensen et al., 2007a). Interestingly, complete abolishment (Dnmt1, Dnmt3a, and Dnmt3b triple knockout) results in abnormal enrichment of H3K27me3 at some loci of pericentric heterochromatin, where it instead causes a delay in replication timing (Takebayashi et al., 2021). However, this redistribution of H3K27me3 does not cause RT changes in other regions of the genome and replication timing at these loci appears to be more dependent on transcriptional changes caused by loss of DNA methylation. In another study, it was demonstrated that H3K27me3 enrichment broadly correlates with mid-S phase replicating origins (Picard et al., 2014).

Monomethylation and dimethylation of lysine 20 of histone H4 (H4K20me1 and me2) are found throughout the genome and coincide with chromatin compaction and transcriptional inactivation, whereas trimethylation (H4K20me3) is exclusively enriched at pericentric heterochromatin and imprinting control regions (Schotta et al., 2004; Karachentsev et al., 2005; Delaval et al., 2007). The methyltransferase PR-Set7 deposits H4K20me1 at replication origins and facilitates loading of the pre-RC complex during licencing. This activity is regulated by ubiquitin-mediated degradation of PR-Set7 during S phase to prevent re-replication (Tardat et al., 2010). In contrast, SET8-dependent methylation of H4K20 has been implicated in repressing replication origin licencing through facilitating chromatin compaction (Shoaib et al., 2018). H4K20me1 is present in approximately half of origins and enriched in early- and mid-replicating regions (Picard et al., 2014). The control of the proportion of nucleosomes with each methylation state of H4K20 is critical to regulate origin selection to ensure no re-replication of DNA, allowing faithful replication of the genome (Schotta et al., 2008; Beck et al., 2012). In addition, H4K20me2 is enriched at replication origins, where it acts as a binding site for ORC1, and H4K20me3 is vital for ensuring the late replication of pericentric heterochromatin (Kuo et al., 2012; Brustel et al., 2017).

#### 2.1.3 Modulation of Replication Timing by Heterochromatin Protein 1

Heterochromatin protein 1 (HP1) is a non-histone protein that is recruited to regions of heterochromatin through binding to methylated H3K9 (Lachner et al., 2001). This factor is an identifier of heterochromatin in several organisms and plays a major role in maintaining the local chromatin environment of heterochromatin. Therefore, the different isoforms of HP1 (HP1α, β and γ in humans) play varying roles in regulating processes including transcription, chromatin compaction (Eissenberg et al., 1990; Kwon and Workman, 2011), cell differentiation (Mattout et al., 2015; Casale et al., 2019) and DNA repair (Dinant and Luijsterburg, 2009; Bártová et al., 2017). In *Drosophila,* HP1 has contrasting roles in replication timing depending on nuclear localisation and H3K9 methylation status. HP1 has been implicated in promoting the early replication timing of some regions with low H3K9me2 enrichment and in mediating later replication of regions with greater H3K9 methylation, such as pericentric heterochromatin (Schwaiger et al., 2010) (Figure 1). Additionally, HP1 is sufficient to induce the late replicating characteristic normally seen in heterochromatic regions when artificially tethered to earlier replicating loci (Pokholkova et al., 2015). Interestingly, HP1 interacts with ORC, a complex that promotes origin firing, and this association is important in HP1α localisation and heterochromatin formation (Pak et al., 1997; Prasanth et al., 2010).

However, mouse HP1 was shown to be dispensable for the maintenance of late replication within pericentric heterochromatin, with the methylation status of H3K9 being more important in establishing the RT of these regions (Wu et al., 2006). Therefore, it is still unclear whether the roles of HP1 in controlling the late replication of pericentric heterochromatin is conserved across organisms or whether H3K9 methylation, a repressive mark which recruits HP1, is more important.

### 2.2 Restoration of Chromatin Following Replication

#### 2.2.1 Formation of Nucleosomes Behind the Fork

During replication, histones must be dissociated from the DNA to allow progression of the replication fork and it is important that they are accurately restored on the parental strand and duplicated onto the daughter strand. Movement of the replisome destabilises chromatin, leading to decondensation and increased mobility of linker histone H1 (Gasser et al., 1996; Contreras et al., 2003; Kuipers et al., 2011). Indeed, mobility of H1 regulated by its phosphorylation is involved in controlling replication timing, presumably by allowing chromatin relaxation (Contreras et al., 2003; Alexandrow and Hamlin, 2005; Katsuno et al., 2009; Thiriet and Hayes, 2009).

Histone deposition is not identical across the leading and lagging strands of replication, and therefore strand-specific mechanisms of chromatin restoration have been identified which are dependent on several components of the replisome. For example, MCM2 and polymerase α directly bind to parental H3-H4 and recycle it onto the lagging strand, and Dpb3 and Dpb4 (accessory subunits of polymerase *ε*) facilitate the deposition of H3-H4 onto the leading strand (Gan et al., 2018; Petryk et al., 2018; Yu et al., 2018; Li et al., 2020). It is important that these strand-specific mechanisms of histone deposition are highly regulated to allow symmetric inheritance of histones, alongside their post-translational modifications, during duplication of the genome.

As the replication fork progresses, parental histones are recycled onto the nascent DNA and newly synthesised histones are added in any gaps with the help of histone chaperones (Alabert and Groth, 2012; Stewart-Morgan et al., 2020). For example, chromatin assembly factor 1 (CAF-1) is an important chaperone which interacts with the replication factor PCNA to deposit histones as replication occurs and has roles in maintaining heterochromatin and transcriptional regulation (Yu et al., 2015). The anti-silencing function 1 (ASF1) chaperone interacts with Mcm2-7 and deposits parental and new H3-H4 dimers directly behind the replication fork in a process that is disrupted during replication stress (Groth et al., 2007; Jasencakova et al., 2010). Another important histone chaperone is called facilitates chromatin transcription (FACT), which also interacts with the replication machinery and has roles in both the eviction and deposition of histones during replication (Formosa, 2012). In order to maintain the epigenomic features of the parental DNA, specific histone variants must also be deposited onto the nascent DNA using specialised histone chaperones. This restoration of chromatin does not always occur in a replication-dependent manner; for example, centromere protein A (CENP-A, a H3 variant) deposition by Holliday junction recognition protein (HJURP) at centromeres and H3.3 deposition by death-associated protein 6 (DAXX) at pericentric and telomeric heterochromatin occur after replication in the following G1 phase (Ahmad and Henikoff, 2002; Jansen et al., 2007; Foltz et al., 2009; Goldberg et al., 2010; Lewis et al., 2010).

#### 2.2.2 Maintenance of Histone Modifications

Following the deposition of nucleosomes onto nascent DNA, it is important that histone PTMs from the parental chromatin are inherited so that the newly synthesised chromatin maintains the same transcriptional regulation, conformation, and replication timing. Methylation and acetylation marks are maintained on parental histones, but these are only present on half of the chromatin following replication due to the incorporation of new unmodified histones, so further processes are required to ensure full restoration of PTMs (Scharf et al., 2009; Xu et al., 2012a; Alabert et al., 2015). If a residue is mono-/dimethylated (e.g., H3K9me1/me2, H3K27me1/me2 and H4K20me1/me2), this mark is diluted following replication and newly incorporated histones obtain these PTMs *de novo* within one cell cycle to become identical to the parental histones (Alabert et al., 2015). On the other hand, trimethylation (e.g., H3K9me3 and H3K27me3) is established slowly and continuously, independent of replication, across several cell divisions (Alabert et al., 2015; Reverón-Gómez et al., 2018). Consequently, oscillations in the levels of histone marks can be observed throughout the cell cycle, and these are not equal at all loci and for all types of histone modification. For example, the H3K4me3 mark enriched at active promoters recovers much quicker than repressive marks such as H3K9me3 and H3K27me3 (Alabert et al., 2015; Reverón-Gómez et al., 2018; Stewart-Morgan, Petryk and Groth, 2020).

#### 2.2.3 Roles of Heterochromatin Protein 1 in Maintaining Heterochromatin

In mouse cells, the interaction of HP1 with the histone chaperone CAF-1, particularly the large p150 subunit, is essential for S phase progression (Quivy et al., 2008). Disruption of this interaction impedes replication of pericentric heterochromatin in a manner that is independent of the canonical histone deposition roles for CAF-1. Intriguingly, p150 is essential for mouse embryo viability during the period of development where HP1-enriched domains are formed (the 8–16 cell stage), suggesting major importance for the p150-HP1 interaction in preserving the survival of cells that are rapidly proliferating and therefore undergoing frequent DNA replication (Houlard et al., 2006).

Additionally, the p150-HP1 complex colocalises with the histone methyltransferase SETDB1 to promote monomethylation of non-nucleosomal histone H3.1 (Loyola et al., 2009). This is important to allow formation of H3K9me1 at newly incorporated histones immediately following DNA replication, which then nucleates formation of trimethylated H3K9 catalysed by Suv39H1/H2. Additionally, the HP1-CAF-1-SETDB1 complex has roles in depositing HP1 at sites of pericentric regions already enriched in H3K9me3. Therefore, CAF-1 has numerous roles in preserving repressive H3K9me3 and HP1 enrichment at pericentric heterochromatin following replication (Loyola et al., 2009).

## 3 Role of the Local Chromatin Environment During Replication Stress

Replication stress (RS) is defined as the slowing or blocking of replication fork progression by a range of endogenous and exogenous sources. Mild RS only results in the slowing of the replication fork velocity and the activation of dormant origins in order to complete replication (Técher et al., 2017). However, as RS gets more severe, the cellular response becomes more intricate. Prolonged RS may lead to uncoupling of the replisome, resulting in production of stretches of replication protein A (RPA)-bound ssDNA which activate the ataxia telangiectasia and Rad3-related (ATR) kinase (Byun et al., 2005). Downstream effectors of ATR such as checkpoint kinase 1 (CHK1) activate the intra-S phase checkpoint which protects the genome from further instability by inhibiting late origin firing and cell cycle progression and promoting DNA repair pathways (Iyer and Rhind, 2017). In a process called fork reversal, the stalled replication fork is remodelled to form a four-way junction to provide protection against excessive degradation (Neelsen and Lopes, 2015). Although this fork reversal, in combination with the recruitment of multiple factors including RAD51 and FANCD2, prevents degradation by nucleases such as MRE11, DNA2 and MUS81, some controlled resection is required to allow rescue of replication (Bryant et al., 2009; Schlacher et al., 2012; Thangavel et al., 2015; Lemaçon et al., 2017). Replication restart following this remodelling of the fork requires the recruitment of several HR proteins, importantly RAD51, which facilitate homology-directed restart (Ait Saada et al., 2018). Therefore, components of homologous recombination, a pathway canonically associated with DSB repair, play various roles in fork remodelling, protection and restart in the event of replication stress. In this section, we will discuss how several aspects of the local chromatin environment are involved in the regulation of these pathways that play central roles in the resolution of replication stress (Table 1).

**TABLE 1:** The roles of histone variants and modifications in replication stress.

Histone variant/modification	Chaperone/enzyme	Functions in replication stress	Reference(s)
H2A.X	—	Phosphorylation at serine-139 (γH2AX) by ATM/ATR is an early marker of damage and involved in repairing replicative lesions in checkpoint-blind yeast	Ward and Chen (2001), Redon et al. (2003), Sirbu et al. (2011), Lyu et al. (2019), Saldivar et al. (2018)
		γH2AX maintains normal S/G2 phase transition during unperturbed replication	
macroH2A	FACT	Promotes HR factor recruitment to stressed forks and persists at fragile sites after replication stress resolution to protect from future replication stress	Kim et al. (2018)
	LSH	Promotes HR factor recruitment to stressed forks	Xu et al. (2021)
		Depletion causes increased H4K20me2, which supresses fork protection	
	—	Protects the inactive X chromosome from replication stress	Sebastian et al. (2020)
H2A.Z (Htz1)	SWR-C	Prevents misincorporation on dNTPs and collapse of stalled replication forks	Van et al. (2015), Srivatsan et al. (2018)
CENP-Α	HJURP	Suppresses formation of centromeric R-loops to prevent TRCs	Giunta et al. (2021)
H2A K13/K15 Ub	RNF168	Facilitates normal S phase progression and promotes fork restart	Schmid et al. (2018); Nakamura et al. (2021)
H2B K123Ub	Bre1 (yeast)	Stabilises nucleosomes on newly replicated DNA an facilitates fork progression	Trujillo and Osley (2012), Lin et al. (2014), Northam and Trujillo (2016), Hung et al. (2017)
		Activates the intra-S phase checkpoint	
		Maintains error-free DNA damage tolerance	
H3K4me1/me3	MLL3/4	Promotes MRE11-depedendent fork degradation in BRCA-deficient cells	Ray Chaudhuri et al. (2016)
	SETD1A	Promotes RAD51-mediated fork protection through H3.1 chaperoning	Higgs et al. (2018)
	Set1 (yeast)	Prevents TRCs in active regions	Chong et al. (2020)
H3K9me2/me3	Met-2, set-25 (*C. elegans*)	Represses genes in repetitive regions to prevent R-loop formation	Zeller et al. (2016)
H3K27me3	EZH2	Recruits MUS81 to facilitate fork restart	Rondinelli et al. (2017)
H4K20me1/me3	PR-Set7	Prevents replication stress by controlling fork number and velocity	Jørgensen et al. (2007a), Tardat et al. (2007), Saredi et al. (2016)
		H4K20me0 on new histones provides binding site for TONSL-MMS22L to facilitate post-replicative repair	
H4 K5/K12 ac	HAT1	Prevents replication stress and protects stressed forks from MRE11-dependent degradation	Nagarajan et al. (2013), Agudelo Garcia et al. (2020)
H4K8ac	PCAF	Promotes MRE11- and EXO1-dependent degradation of stalled forks in BRCA2-deficient cells	Kim et al. (2020)

### 3.1 Histone Variants

In humans, there are several variations of histones H2A, H2B, H3, and H4 which have different sequences to their canonical histone counterparts and may be localised to specific genomic regions to influence the structure and function of the chromatin (Martire and Banaszynski, 2020). Here, we will describe the importance of some of these histone variants during replication and particularly in the event of replication stress.

#### 3.1.1 Histone H2AX

The histone H2A variant H2A.X has major implications in genome stability: specifically, H2A.X phosphorylated at serine-139 rapidly after DSB induction, creating a mark known as γH2AX (Rogakou et al., 1998). γH2AX formation catalysed by the ATR signalling cascade also occurs following replication stalling before the collapse of forks in DSBs, although inhibition of Chk1, a downstream factor of ATR, has been shown to induce γH2AX formation (Ward and Chen, 2001; Sirbu et al., 2011). This association of γH2AX presumably facilitates recruitment of DNA repair proteins and studies in yeast have revealed a role in repairing replicative damage in cells without intra-S phase checkpoint activation (Redon et al., 2003). Interestingly, γH2AX distribution following replication stress is not equal across the genome and greater association is seen in commonly fragile regions containing compact chromatin which are depleted of transcription start sites and CpG islands (Lyu et al., 2019). Therefore, this modification could be primarily important to promote certain pathways involved in the resolution of replicative damage in specific chromatin contexts, or this enrichment could reflect persistent replication stress particularly in fragile regions of the genome that are difficult to repair. In addition, ATR kinase activity and H2AX phosphorylation also occur during unperturbed S phase to facilitate the correctly timed transition into G2 phase (Saldivar et al., 2018). Consequently, γH2AX not only has direct roles during stressed replication but also is essential to maintain genome integrity by preventing premature entry into mitosis and under-replication of the genome.

#### 3.1.2 Macro H2A

MacroH2A is a subfamily of histone H2A variants comprised of three isoforms called macroH2A1.1, macroH2A1.2 (which are splice variants from the same gene) and macroH2A2 (Rasmussen et al., 1999; Costanzi and Pehrson, 2001). Structurally, these proteins are composed of a H2A domain with an N-terminal non-histone macro domain and are approximately three times the size of other H2A variants (Pehrson and Fried, 1992). Some functions of this histone variant have been linked to X chromosome inactivation (Costanzi and Pehrson, 1998; Mermoud et al., 1999; Rasmussen et al., 2000), transcriptional regulation (Ouararhni et al., 2006; Gamble et al., 2010; Creppe et al., 2012) and nucleosome organisation (Angelov et al., 2003; Abbott et al., 2004; Chakravarthy and Luger, 2006; Muthurajan et al., 2011; Chakravarthy et al., 2012). Additionally, macroH2A is enriched at heterochromatin regions, particularly those marked by H3K9me3, and has roles in higher genome organisation (Douet et al., 2017; Kozlowski et al., 2018).

Alongside functions in homology-directed DSB repair (Xu et al., 2012b; Khurana et al., 2014), macroH2A1.2 also has roles during replication stress, where it is deposited onto chromatin by the FACT histone chaperone (Kim et al., 2018). This activity is particularly important at common fragile sites (CFS), regions that are more prone to replication stress induced damage. MacroH2A deposition is also assisted by the LSH chromatin remodeller and promotes BRCA1 and RAD51 recruitment to stalled forks to promote fork protection and facilitate repair (Kim et al., 2018; Xu et al., 2021). Recently, it was shown that loss of this macroH2A deposition is associated with an increase of H4K20me2 at stalled forks which then favours 53BP1 recruitment to stalled forks rather than BRCA1, causing a detrimental effect on fork protection (Xu et al., 2021). MacroH2A1.1 and 1.2 are enriched on the mammalian female inactive X (Xi) chromosome, a highly condensed genomic region with increased susceptibility to replication stress (Costanzi and Pehrson, 1998; Koren and McCarroll, 2014). The macroH2A1.2 variant is involved in supressing replication stress at the Xi, whereas the splice variant macroH2A1.1 activates the alternative end joining DSB repair pathway, leading to Xi anaphase defects in the absence of H2A1.2 (Sebastian et al., 2020). Overall, these studies suggest an importance for macroH2A in facilitating the proper repair of difficult-to-replicate genomic loci such as CFS’s and the inactive X chromosome. The replication stress-dependent recruitment of macroH2A to fragile sites is mainly transient but some remains after resolution of replication stress and protects these difficult to replicate regions from future replicative damage (Kim et al., 2018). Since macroH2A has roles in repressing gene expression, X inactivation and nucleosome organisation, it would be important to see whether this continued enrichment alters transcription and genome organisation at these sites.

#### 3.1.3 H2A.Z

H2A.Z is a variant of histone H2A that is incorporated throughout the cell cycle and has the ability to alter the physical properties of the nucleosome and create specialised chromatin structures (Fan et al., 2002, 2004). This histone variant has conserved functions in the regulation of transcription in euchromatic genes (Zhang et al., 2005; Hardy et al., 2009). Nucleosomes associated with H2A.Z also enhance replication origin firing efficiency through promoting H4K20 dimethylation and ORC1 recruitment (Long et al., 2020). In yeast, incorporation of the H2A.Z homologue Htz1 by Swi2/Snf2-related chromatin remodelling complex (SWR-C) is important in maintaining genome stability following both DSBs (Kalocsay et al., 2009; Horigome et al., 2014) and replication stress (Van et al., 2015; Srivatsan et al., 2018). Specifically, Hitz1 has roles in preventing the misincorporation of nucleotides during replication and in promoting the repair of replicative damage (Van et al., 2015; Srivatsan et al., 2018). Incorporation of Htz1 during replication stress has been hypothesised to prevent collapse of forks into DSBs by two mechanisms: either it stabilises the fork to prevent replisome dissociation, or it is incorporated after replisome dissociation to prevent further degradation of the fork by nucleases (Srivatsan et al., 2018). Furthermore, the balance of H2A.Z on chromatin is vital: as removal by the INO80 chromatin remodeller is also important in maintaining replication fork stability and to allow HR to occur (Papamichos-Chronakis et al., 2011; Lademann et al., 2017).

#### 3.1.4 CENP-Α at Centromeres

Despite being located between pericentromeric regions expressing features of heterochromatin, mammalian centromeres display active epigenetic marks (e.g., H3K4me2 and H3K36me2) combined with production of long non-coding RNA transcripts (Sullivan and Karpen, 2004). In human cells, centromeres are composed of tandem alpha satellite repeats and enriched in CENP-A, a histone H3 variant specific for centromeres which has key roles in formation of kinetochores to allow proper chromosome segregation (Barra and Fachinetti, 2018). Because of their involvement in chromosome segregation, the maintenance of centromere integrity during replication is vital to ensure proper cell division.

Unlike many canonical histone variants, CENP-Α is not deposited onto newly replicated DNA during S-phase but is instead incorporated in the following G1-phase by its dedicated histone chaperone, HJURP (Jansen et al., 2007). Contrary to this, CENP-A has recently been shown to possess key roles in maintaining centromere integrity during DNA replication (Giunta et al., 2021). CENP-A deposition prevents the formation of centromeric R-loops formed as a consequence of transcription-replication conflicts (TRCs) during late S phase, thereby promoting fork progression. Centromeres depleted of CENP-A display multiple replication-associated defects such as error-prone mitotic DNA synthesis (MiDAS) and chromosomal translocations, breakages, and fragmentation. These chromosomal aberrations are the result of recombination between alpha satellite repeats in an R-loop dependent manner rather than being due to defects in chromosomal segregation during mitosis (Giunta et al., 2021). The specialised function for CENP-A in removing R-loops is important because some alpha-satellite RNA transcripts remain associated with the centromere and could therefore disrupt the progression of incoming replication forks (McNulty et al., 2017).

### 3.2 Role of Linker Histone H1

In human cells, eviction of the linker histone H1 by the histone chaperone SET results in sensitivity to DNA damage (Mandemaker et al., 2020). Although depletion of SET does not alter normal S phase progression, it does cause resistance to the replication stress-inducing agent HU; suggesting important roles for chromatin-bound H1 during perturbed replication (Mandemaker et al., 2020). In agreement, overexpression of SET causes slower S phase progression upon treatment of HU (Kalousi et al., 2015). SET also promotes retention of the heterochromatin factors KAP1 and HP1 on chromatin which causes chromatin compaction and inhibition of HR repair of collapsed forks (Kalousi et al., 2015). Potentially, loss of SET and subsequent H1 retention and chromatin decompaction could allow for greater access of DNA repair factors to replicative lesions, thereby promoting proper repair and cell survival.

In addition, the linker histone dH1 in *Drosophila* has been implicated in preventing the accumulation of R-loops, a structure which can induce replication stress (Bayona-Feliu et al., 2017). Upon depletion of dH1, transcriptional repression is relieved within heterochromatin regions, leading to the accumulation of R-loops. Interestingly, R-loop accumulation was not seen upon depletion of HP1a, suggesting that this effect is only seen upon loss of H1 and is not a general effect of transcriptional derepression in heterochromatin (Bayona-Feliu et al., 2017), although loss of repression in repetitive regions has been associated with R-loop prevention in *C. elegans* (Zeller et al., 2016). Notably, in mouse cells depleted of histone H1, fork stalling as a result of transcription-replication conflicts has also been observed (Almeida et al., 2018). H1 loss leads to chromatin decompaction which causes acceleration of both transcription initiation and replication leading to pathogenic accumulation of R-loops and collisions between the transcription and replication machineries (Almeida et al., 2018).

The linker histone H1 has been shown to have roles in both preventing the onset of replication stress and in facilitating the repair of replicative damage. Currently, it is unclear how H1 elicits these effects: whether it is due to its roles in organising nucleosome particles into stable higher order structures and maintaining proper spacing between nucleosomes or in the formation of heterochromatin and maintaining transcriptional repression (Happel and Doenecke, 2009).

### 3.3 Histone Modifications and Replication Stress

#### 3.3.1 Histone Ubiquitination

Ubiquitination of histone H2AK13 and K15 adjacent to DNA lesions plays a central role in the DNA damage response, where these marks facilitate the recruitment of several DNA repair factors (Smeenk and Mailand, 2016). During unchallenged S phase, H2A ubiquitination by RNF168 and activation of the DNA damage response (DDR) is required for normal replication progression and to prevent fork stalling (Schmid et al., 2018). In addition, RNF168 plays a role in preventing accumulation of reversed forks through restarting stalled replication, particularly at difficult to replicate repetitive regions of the genome (Schmid et al., 2018). Formation of γH2AX by ATR and ATM kinases occurs upstream of H2A ubiquitination and is involved in coordinating this DDR activation during S phase (Schmid et al., 2018; Nakamura et al., 2021). Interestingly, ATM inhibition increases activation of the histone ubiquitin response upon fork breakage by camptothecin. This is possibly because ATM is involved in a negative feedback loop alongside PLK1 where end resection at single-ended DBSs deactivates the histone ubiquitination pathway (Nakamura et al., 2021).

Monoubiquitinated histone H2B is a dynamic histone mark first associated with transcription and repair (Kao, 2004; Fleming et al., 2008) also possessing other roles in controlling chromatin compaction during DSB repair (Moyal et al., 2011; Nakamura et al., 2011). In yeast, the ubiquitin ligase responsible for H2B monoubiquitination, Bre1, is maintained on replicating DNA and is further enriched at stressed replication forks (Trujillo and Osley, 2012; Lin et al., 2014; Hung et al., 2017). During replication, H2B ubiquitination plays a role in regulating nucleosome assembly onto replicating DNA, which facilitates normal fork progression and replisome stability during replication stalling by HU (Trujillo and Osley, 2012). In contrast, H2Bub has also been shown to restrict fork progression upon HU-induced stress through coordinating chromatin assembly and activation of the Rad53-dependent intra-S checkpoint (Lin et al., 2014). Intriguingly, other roles for H2Bub during replication stress are linked to its involvement in regulating DNA damage tolerance (DDT) pathways (Northam and Trujillo, 2016; Hung et al., 2017). This mark promotes polymerase eta (Polη)-dependent translesion synthesis (TLS) and suppresses the more mutagenic polymerase zeta (Polζ)-dependent pathway following fork stalling by HU and UV treatment (Northam and Trujillo, 2016). In another study, monoubiquitinated H2B was shown to promote recombination-dependent lesion bypass following treatment with alkylating agents, specifically by altering the chromatin dynamics and allowing RAD51 recruitment (Hung et al., 2017). This mark then facilitates repair of these bypassed lesions post-replication, possibly by promoting activation of the G2/M checkpoint. Therefore, this histone modification has cell cycle-specific roles for maintaining genome replication following replicative damage checkpoint (Hung et al., 2017). Whilst these studies have elucidated the importance of H2B monoubiquitination and chromatin dynamics during replication stress, it remains unclear how this modification has seemingly contradictory roles in both stalling of replication through checkpoint activation and coordinating DDT pathways to allow DNA synthesis beyond lesions and subsequent post-replicative repair.

#### 3.3.2 Histone H3 Modifications

Methylation of H3K4 has been implicated to have contrasting roles in the protection of stalled replication forks in mammalian cells depending on which methyltransferase deposits this mark (Ray Chaudhuri et al., 2016; Higgs et al., 2018). In mouse cells, MLL3/4 is responsible for depositing H3K4me1 and H3K4me3 at stressed forks, where it promotes fork degradation by MRE11 in BRCA-deficient mouse cells with existing defects in fork protection (Ray Chaudhuri et al., 2016). Therefore, loss of H3K4 methylation by MLL3/4 rescues fork protection in BRCA-deficient cells, leading to chemoresistance (Ray Chaudhuri et al., 2016). In contrast, monomethylation of H3K4 at stressed forks by human SETD1A promotes RAD51-dependent replication fork protection from degradation by DNA2 and CHD4 (Higgs et al., 2018). This fork protection is mediated through chaperoning of histone H3.1 by the Fanconi anaemia factor FANCD2 (Higgs et al., 2018), a factor involved in interstrand crosslink repair, RAD51-mediated replication fork protection and the replication of fragile sites (Schlacher et al., 2012; Sato et al., 2012; Madireddy et al., 2016; Higgs et al., 2018). It is interesting that methylation of H3K4 by different methyltransferases leads to opposite effects on fork protection and this could mean that these enzymes are either active at different times during the replication stress response, are recruited differentially to certain genomic loci or are responsible for methylation within a specific local chromatin context (e.g., presence of H2BUb) (Wu et al., 2008; Higgs et al., 2018). Additionally, H3K4me is commonly found in active regions, so it would be important to investigate whether these mechanisms are mostly important at these regions or whether the mark is added *de novo* throughout the genome in response to replication stress. Notably, fork protection upon loss of MLL3/4 was observed only in BRCA-deficient cells whereas fork degradation by SETD1A loss was observed in BRCA-WT cells (Ray Chaudhuri et al., 2016; Higgs et al., 2018). Therefore, the promotion of fork degradation by MLL3/4 mediated methylation of H3K4 may specifically occur when HR-mediated fork protection is lost.

In yeast, H3K4 methylation plays a role in slowing down fork progression through highly transcribed regions to prevent transcription-replication conflicts, thereby ensuring faithful replication (Chong et al., 2020). Indeed, transcriptionally active genes are commonly decorated with H3K4me3 (Santos-Rosa et al., 2002; Guenther et al., 2007). Another histone H3 modification with roles in preventing TRCs is methylated H3K9 (Zeller et al., 2016), a mark commonly enriched at heterochromatin that plays a role in HP1 recruitment and transcriptional repression (Bannister et al., 2001; Lachner et al., 2001; Nakayama, 2001). In *C. elegans*, H3K9me2/me3 function to repress genes in repetitive elements, thereby suppressing R-loop formation and preventing replication stress (Zeller et al., 2016).

Trimethylated H327 is also a mark of transcriptionally inactive chromatin and is deposited by enhancer of zeste homologue 2 (EZH2), a component of the polycomb repressive complex 2 (PRC2), during both G1 and S phases (Hansen et al., 2008; Morey and Helin, 2010). Alongside its important roles in transcription and cell identity (Wiles and Selker, 2017), H3K27me3 also plays a role during replication stress (Rondinelli et al., 2017). Methylated H3K27 is involved in the recruitment of MUS81 to stalled forks, an endonuclease which creates DSBs to facilitate HR-mediated fork restart (Hanada et al., 2007; Rondinelli et al., 2017). Whilst activation of this pathway is required for replication restart in BRCA2-deficient cells, excessive fork degradation by MUS81 can be toxic. Therefore, in a similar manner to with the MLL3/4/H3K4me/MRE11 axis (Ray Chaudhuri et al., 2016), loss of EZH2 and subsequent fork protection from MUS81 endonuclease activity leads to chemoresistance in BRCA-deficient cells (Rondinelli et al., 2017). These roles of epigenetic modifications in protecting the replication fork, thereby allowing cancer cells to become resistant to therapies, have major clinical implications. For example, expression levels of EZH2 could be used as a biomarker to predict resistance to drugs such as poly (ADP-ribose) polymerase (PARP) inhibitors and allow more stratified treatment for BRCA-deficient cancers (Rondinelli et al., 2017).

#### 3.3.3 Histone H4 Modifications

In addition to its roles in replication timing, the methyltransferase PR-Set7 responsible for H4K20 methylation has roles in facilitating S phase progression and during replication stress, where it interacts with the key replication factor PCNA (Jørgensen et al., 2007b). In addition, depletion of this factor affects the number and velocity of replication forks and causes activation of p53 and Chk1 dependent checkpoints. Loss of H4K20 methylation in this manner increases the frequency of replicative DNA breaks which then recruit repair factors such as RPA, RAD51 and 53BP1 (Jørgensen et al., 2007b; Tardat et al., 2007). Importantly, unmethylated H4K20 which marks newly incorporated histones plays a role in regulating the post-replicative repair of DNA lesions obtained during S phase (Saredi et al., 2016). H4K20me0 provides a binding site for TONSL-MMS22L in G2/M phases, and this complex then promotes HR repair (Duro et al., 2010; Saredi et al., 2016). Overall, these studies show roles for H4K20 methylation status in the regulation of replication fork progression and, in addition, the repair of replicative lesions after S phase completion.

Histone acetyltransferase 1 (HAT1) catalyses the acetylation of histone H4 at lysine 5 and lysine 12 and this activity is important in the processing of newly deposited histones during replication (Nagarajan et al., 2013). Depletion of HAT1 results in replication stalling and sensitivity of cells to replication stress induced by HU (Nagarajan et al., 2013; Agudelo Garcia et al., 2020). The importance of HAT1 in maintaining genome stability during replication stress is due to its roles in protecting stalled forks from degradation by MRE11 (Agudelo Garcia et al., 2020). In contrast, acetylation of H4K8 by p300/CBP-associated factor (PCAF) promotes nucleolytic degradation of stalled forks in BRCA-deficient cells, suggesting differential roles for histone acetylation on replication fork protection depending on which residue is modified and by which acetyltransferase (Kim et al., 2020). Acetylation of H4K8 by PCAF provides a binding site for MRE11 and EXO1 nucleases, and this activity is suppressed by ATR-mediated phosphorylation of PCAF to maintain tight control of stalled fork degradation. In BRCA2-deficient tumours where fork protection is compromised, low levels of PCAF activity is associated with resistance to PARP inhibition through restoring fork protection (Kim et al., 2020).

### 3.4 HP1 During Replication Stress

In a more recent study, HP1β has been shown to have some important roles during normal replication progression and in the presence replication stress in mammalian cells. Knockout of HP1 in mouse embryonic fibroblasts (MEFs) led to reduced cell growth and fork speeds in combination with enhanced formation of DNA damage foci (Charaka et al., 2020). Upon depletion of deoxyribonucleotides, both mouse and human (Hela) cells depleted of HP1 displayed increased fork stalling and defective fork restart. In support for key roles of HP1 during replication stress, depletion also sensitises human and mouse cells to HU and cisplatin, resulting in increased levels of chromosomal aberrations (Charaka et al., 2020). It is currently unknown exactly how HP1 plays a role in the cellular response to replication stress, however it could share some similarities with its roles in DSB repair. For example, HP1 depletion leads to reduced recruitment of BRCA1 to DSBs, so perhaps the importance of HP1 in the resolution of replication stress is dependent on an ability to recruit BRCA1, which then promotes RAD51-dependent fork protection (Schlacher et al., 2012; Lee et al., 2013). On the other hand, HP1 retention on heterochromatin has been implicated in *preventing* homology-directed repair of heterochromatic DSBs and mobilisation of HP1β plays a role in the activation of DDR signalling (Ayoub et al., 2008; Kalousi et al., 2015). Currently, it is unclear which specific pathways HP1 plays a role in to resolve replication stress and whether it is strictly important for RS in heterochromatin or whether it has global roles.

## 4 Movement of Stressed Replication Forks

The mobility of DNA lesions, namely DSBs, has been well documented and has been shown to be vital for proper repair in specific circumstances. In mammalian cells for example, whilst heterochromatic DSBs are positionally stable in G1 and are repaired by non-homologous end joining (NHEJ), they are relocated to the periphery of heterochromatin domains during S and G2 phases to allow recruitment of HR factors (Tsouroula et al., 2016). The mobility of DSBs is a conserved response that allows formation of the RAD51 filament and repair of DSBs, especially within heterochromatin, and SUMOylation is an important post translational modification which controls this process (Oza et al., 2009; Ryba et al., 2010). Therefore, certain nuclear compartments provide a protective environment for DNA repair, and this is also important in the event of replication stress. In this section we will discuss how some stressed forks do not remain positionally stable and are relocated to specific nuclear compartments in a similar manner to DSBs.

### 4.1 The Nuclear Pore Complex and Replication Stress

#### 4.1.1 Importance in Repetitive vs. Non-repetitive Sequences

During S phase in yeast, replication stress prone expanded CAG repeats relocate to the nuclear periphery where they interact with components of the nuclear pore complex (NPC) to prevent chromosomal breakages (Su et al., 2015). For this movement to occur, the repair proteins RPA, Rad59 and Rad52 are SUMOylated by Mms21 SUMO E3 ligase which permits their interaction with the SUMO interacting motif (SIM) of Slx5 (Whalen et al., 2020). SUMOylation of the ssDNA-binding protein RPA inhibits Rad51 binding at the stalled fork and this inhibition is lost when collapsed forks associate with NPCs, possibly by degradation of SUMOylated proteins promoted by the SUMO-targeted ubiquitin ligase (STUbL) Slx5/8 (Su et al., 2015; Whalen et al., 2020). Therefore, homology-directed fork restart is suppressed until movement of these repetitive sequences to the nuclear pore. Intriguingly, in a study where replication stress was induced at a unique sequence in yeast, RAD51 binding and activity were able to occur before anchoring to the NPC (Kramarz et al., 2020). Here, SUMOylation by Pli1 was shown to promote fork mobility, and anchoring of the fork to the NPC caused removal of the SUMO chains and allowed recombination-dependent restart. Alternatively, recombination-dependent restart could still even occur without NPC anchoring when SUMOylation by Pli1 was selectively inhibited (Kramarz et al., 2020).

**FIGURE 2:**
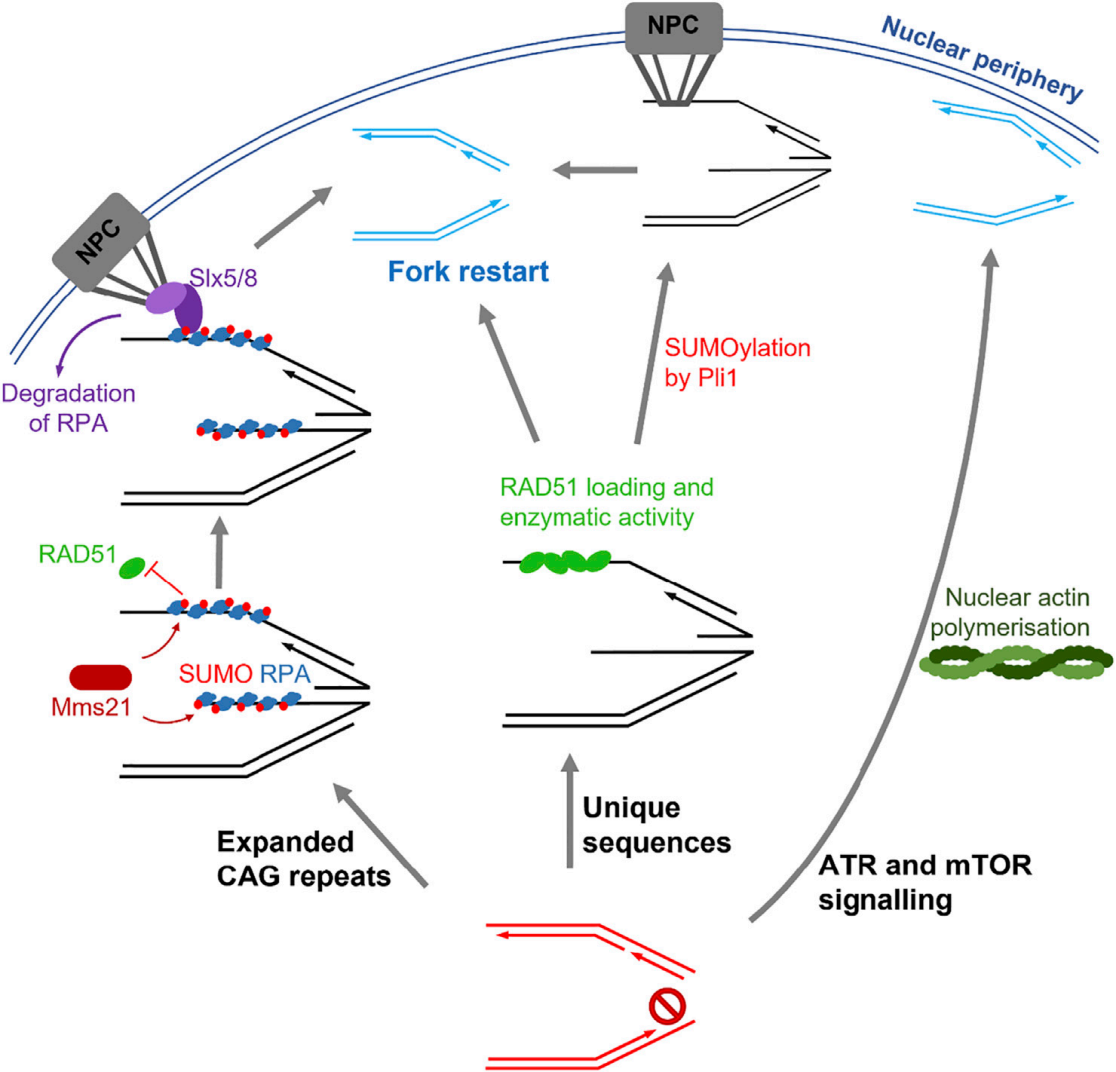
Summary of the pathways involved in stressed fork mobility. Left: fork stalling induced by expanded CAG repeats leads to SUMOylation of repair proteins such as RPA by Mms21, which block RAD51 binding. Tethering of the fork to the nuclear pore complex (NPC) by Slx5/8 binding releases this inhibition and allows fork restart. Centre: Replication fork stalling within unique sequences results in loading of RAD51 in the nucleoplasm followed by SUMOylation events by Pli1, which allow NPC tethering and fork restart. Alternatively, fork restart can also occur in the absence of SUMOylation and NPC tethering. Right: ATR and mTOR signalling during replication stress leads to polymerisation of nuclear actin. Stalled forks then move along the actin filaments to the nuclear periphery, where fork restart can occur.

Overall, these studies suggest that different mechanisms of replication stress resolution occur depending on the nature of the DNA sequence (Figure 2). In repetitive sequences (i.e., expanded CAG repeats), RAD51 binding is limited until tethering to the NPC, which is essential to allow replication restart and prevention of DSBs (Su et al., 2015; Whalen et al., 2020). Conversely, within non-repetitive sequences, RAD51 activity occurs at stalled forks before relocation to the NPC, and restart can occur with or without NPC tethering (Kramarz et al., 2020). Therefore, mobility of stalled forks and tethering to the NPC may only be necessary for repetitive sequences, which are likely to undergo detrimental recombination events if in close proximity to other repetitive sequences. The involvement of SUMO is similar in both repetitive and unique sequences, where it promotes mobility of stalled forks but is removed upon tethering to the NPC for replication restart to occur (Su et al., 2015; Kramarz et al., 2020; Whalen et al., 2020).

#### 4.1.2 Anchoring of Stressed Telomeres to the Nuclear Pore Complex

Telomeres in yeast and human cells are relocated to the nuclear pore complex in response to genomic stress (Khadaroo et al., 2009; Churikov et al., 2016; Pinzaru et al., 2020). In budding yeast, this relocalisation of eroded telomeres is SUMO- and STUbL-dependent and promotes type II recombination [the mechanism of alternative lengthening of telomeres (ALT) in yeast] (Churikov et al., 2016). In human cancers, replication stress at telomeres can be arise due to dysfunction of the telomere protection factor POT1 (Pinzaru et al., 2016; Pinzaru et al., 2020). This type of replicative stress causes increased MiDaS at telomeres and relocation of a small fraction of them to nuclear pore. This movement is promoted by polymerisation of nuclear actin and is required to maintain telomere integrity in POT1-deficient cells. Indeed, disruption of the nuclear pore complex in cells harbouring a DNA binding deficient POT1 mutant exacerbates telomere repeat instability and detrimental telomeric recombination events (Pinzaru et al., 2020).

#### 4.1.3 Processing of R-Loops at the Nuclear Pore Complex

Several studies have implicated roles for the NPC in the resolution of transcription-replication conflicts. It has been hypothesised that transcribed genes are transiently localised to the nuclear pores to aid in nuclear export of transcription products in a process called gene gating (Blobel, 1985). In yeast, localisation of transcribed genes to the NPC is important in preventing pathological R-loop formation, suggesting that gene gating and the subsequent nuclear transport of nascent RNA is important in preventing TRCs (García-Benítez et al., 2017). The human NPC component Tpr has also been implicated in the processing of DNA-RNA hybrids (Kosar et al., 2021). Interestingly, activation of the ATR-dependent checkpoint during replication stress releases transcribed genes from the nuclear pore to facilitate fork restart (Bermejo et al., 2011). These results suggest that proximity to the nuclear pore is important in preventing R-loop-dependent replication stress within transcribed genes, but upon exposure to other sources of replication stress, these genes are released from the NPC possibly to *allow* R-loop formation which consequently promotes DDR signalling and fork restart (García-Benítez et al., 2017). In addition, topoisomerases have been shown to suppress RNA-DNA hybrid formation during replication through modulating gene topology (Tuduri et al., 2009; Achar et al., 2020). Specifically, Top2 maintains negative supercoiling at gene boundaries, which facilitates normal transcription and suppresses hybrid formation specifically during S phase (Achar et al., 2020).

### 4.2 Mobility of Stressed Replication Forks by Nuclear Actin

Whilst it remains unclear whether stressed fork mobility is caused by passive diffusion or involves an active mechanism, recent studies have shown that the contractile fibre actin has roles during replication stress, suggesting fork movement is an active process. Transient polymerisation of actin, forming filamentous actin (F)-actin, occurs in the nucleus and is involved in the relocation of DSBs, particularly within heterochromatin, to facilitate HR repair (Belin et al., 2015; Caridi et al., 2018; Schrank et al., 2018). During unperturbed replication, nuclear actin polymerisation is important to allow recruitment of replication factors and S phase progression (Parisis et al., 2017). Additionally, upon replication stress, F-actin formation at stressed replication foci reversibly increases in an ATR and mTOR signalling-dependent manner (Lamm et al., 2020). Stalled forks then move along the F-actin, facilitated by myosin motor proteins, to localise with the nuclear periphery. This localisation allows replication restart, and therefore resolution of replication stress before collapse into a single ended DSB, which distinguishes this pathway from the movement of DSBs to allow HR (Schrank et al., 2018; Lamm et al., 2020) (Figure 2). In addition, the nuclear volume increases as a result of F-actin formation, possibly due to chromatin decompaction, and this response is not seen during DSB repair (Lamm et al., 2020). The movement of stalled forks to the nuclear lamina may allow direct interactions with the nuclear envelope and be important in the recruitment of repair factors; indeed, the laminA/C proteins has been implicated in promoting the recruitment of RPA, RAD51 and FANCD2 (Singh et al., 2013). On the other hand, DSBs located at the nuclear periphery within LADs suppress HR by inhibiting the recruitment of RAD51 (Lemaître et al., 2014). In the future, it will be therefore important to understand the roles of the nuclear lamina in the regulation of homologous-directed repair of DSBs and stalled replication forks.

## 5 Conclusion and Future Perspectives

The organisation of the genome plays roles in facilitating progression of DNA replication on multiple levels. At the higher levels of genome organisation, arrangement of genetic material into higher order structures and localisation within specific compartments play a major role in controlling the replication timing program. In addition, the composition of the nucleosome as defined by histone variants and PTMs, and the enrichment of non-histone proteins all modulate replication timing (Figure 1). In many cases, this replication timing is strongly linked to transcription where active regions correlate with early replication and inactive regions with late. The importance of this link between transcription and replication timing and whether there is any causality between these two processes are still unclear. Possibly, the compacted state of repressed chromatin may act as a barrier to early replication, localisation of inactive/active regions at specific loci may impact origin firing or it may be favourable to duplicate replication stress prone sequences (e.g., gene-poor repetitive regions) later in S phase.

In addition, the chromatin environment plays important roles in the resolution of replication stress. Particularly, the presence of histone variants and modifications at stressed loci play major roles in promoting or repressing specific repair pathways (Table 1). However, the question remains whether these variants/marks are deposited *de novo* at all genomic regions during replication stress and remain at these loci after the resolution of replication stress to protect against future challenges or whether they are present only at specific regions that are more difficult to repair.

The SUMO-directed movement of stressed replication forks to facilitate recombination-dependent repair shares some striking similarities with the mobility of DSBs to the nuclear periphery. This mobility of stalled forks is primarily important within repetitive regions such as expanded CAG repeats and telomeres, and the mobility of DSBs occurs when breaks are situated in heterochromatin (Figure 2). The movement of these specific regions to the nuclear periphery could be important for at least two reasons: movement may prevent detrimental recombination events between repeats and/or the environment of the nuclear periphery may provide a protective environment for the repair of these difficult sequences. Indeed, association of stressed forks with components of the nuclear pore complex is vital for repair of replicative lesions. Future studies linking the roles of nuclear actin and movement of stressed forks to the NPC would be beneficial to understand how these fork movements are regulated and propagated.

Tumours are highly prone to replication stress due to their uncontrolled proliferation and deregulated cellular signalling, and this feature is already being targeted in several therapies in clinic (Ubhi and Brown, 2019). Consequently, some of the features of the chromatin could be exploited to provide novel targeted therapies for cancer. Histone marks that regulate the protection of stalled forks form nucleolytic degradation such as methylated H3K4 and H3K27 and acetylated H4K8 have major implications in the treatment of cancers, specifically those harbouring BRCA1/2 mutations. Therefore, measuring the expression of the enzymes that deposit/remove these marks and the enrichment of the marks themselves could provide biomarkers that could be used in the future to predict resistance of tumours to certain therapies and allow greater stratification of treatment. In addition, drugs that target specific enzymes and modify the epigenome and sensitise cells to other therapies. For example, histone deacetylase (HDAC) inhibitors are already used in the clinic and combination of these with other anti-cancer drugs are being explored (Suraweera et al., 2018). Finally, the movement of stalled forks by nuclear actin is mediated through ATR and mTOR signalling, so these pathways provide attractive targets for anti-cancer drugs. Indeed, polymerisation of nuclear actin facilitates survival of cancer cells (Lamm et al., 2020). In summary, future studies elucidating the role of chromatin during replication stress are important in discovering new and more stratified cancer treatments.
